# COVID-19 restrictions: experiences of immigrant parents in Toronto

**DOI:** 10.3934/publichealth.2021013

**Published:** 2021-02-05

**Authors:** Sepali Guruge, Paula Lamaj, Charlotte Lee, Charlene Esteban Ronquillo, Souraya Sidani, Ernest Leung, Andrew Ssawe, Jason Altenberg, Hasina Amanzai, Lynn Morrison

**Affiliations:** 1 Daphne Cockwell School of Nursing, Ryerson University, Toronto, Canada; 2 South Riverdale Community Health Centre, Toronto, Canada; 3 Department of Anthropology, University of Hawai'i at Hilo, Hilo, Hawai'i, USA

**Keywords:** Canada, coronavirus, COVID-19, immigrant, parenting, physical distancing

## Abstract

Parenting is a demanding undertaking, requiring continuous vigilance to ensure children's emotional, physical, and spiritual well-being. It has become even more challenging in the context of COVID-19 restrictions that have led to drastic changes in family life. Based on the results of a qualitative interpretive descriptive study that aimed to understand the experiences of immigrants living in apartment buildings in the Greater Toronto Area, Ontario, Canada, this paper reports the experiences of 50 immigrant parents. During the summer and fall of 2020, semi-structured interviews were conducted by phone or virtually, audio-recorded, then translated and transcribed. The transcripts were analyzed using thematic analysis. Results revealed that parenting experiences during the pandemic entailed dealing with changing relationships, coping with added burdens and pressures, living in persistent fear and anxiety, and rethinking lifestyles and habits. Amid these changes and challenges, some parents managed to create opportunities for their children to improve their diet, take a break from their rushed lives, get in touch with their cultural and linguistic backgrounds, and spend more quality time with their family. While immigrant parents exhibit remarkable resilience in dealing with the pandemic-related meso and macro-levels restrictions, funding and programs are urgently needed to support them in addressing the impact of these at the micro level.

## 1. Introduction

Being a parent is a rewarding yet stressful experience involving multiple demands. In 2020, parents around the world encountered one of the most difficult challenges to date, the COVID-19 pandemic caused by the rapid transmission of SARS-CoV-2, a new strain of coronavirus. Early in the pandemic in February to March 2020, the Canadian federal government announced physical distancing measures that corresponded to those outlined by the World Health Organization. These measures required families to refrain from having contact with those who did not reside in the same household, and to maintain a physical distance of at least two metres from one another indoors and outdoors. Many jobs were lost, homes became workplaces, brick and mortar schools turned into virtual schools, childcare facilities were closed, and families were confined to their homes. School closures forced children to stay at home 24 hours a day, 7 days a week, and to study at home under the supervision of parents while being physically isolated from their peers, friends, extended family members, and neighbours. Parents who lost their jobs were unable to access paid educational and support services. News and social media have featured many of these parental struggles but thus far, there has not been any published research on the parenting experience among immigrants in Canada that documents these challenges and the responses to them. This paper explores the COVID-19 related changes and challenges experienced by immigrant parents in the Greater Toronto Area of Ontario Canada.

Past research has identified numerous factors associated with parental stress and burnout including parental unemployment, financial insecurity, low levels of social support, and a lack of leisure time [1],[2]. Responses to the pandemic including lockdowns, cancelled services, and other public health measures have magnified many of these stressors. Emerging research has identified the emotional and practical demands of working at home (and at times longer than usual hours), childrearing for 24 hours a day, 7 days a week, restriction of access to public spaces, and balancing the demands of paid employment with the increased responsibilities from having children at home [3]. Studies from countries such as Singapore show that the extreme financial and health insecurity, along with physical and social isolation because of COVID-19, can increase tensions in the home environment [4]. Many parents are finding it extremely difficult to secure time for themselves and to achieve balance between their personal, professional, and parental roles and responsibilities [1].

Recent evidence also reveals the gendered effects of challenges and stressors associated with the pandemic, which are disproportionately experienced by mothers [5],[6]. During the first month of the lockdown, for example, women in the United Kingdom spent on average two-thirds more time on childcare responsibilities than men; in addition, mothers were interrupted much more often than fathers, greatly affecting their ability to work from home during the pandemic [3]. The requirement to stay at home affected mothers' independence, skill acquisition, and feeling of purpose often associated with paid employment outside the home, resulting in isolation, suffocation, boredom, and feelings of oppression and imprisonment [3].

Emerging research also indicates that the pandemic has exacerbated not only gendered inequalities but also existing social, financial, cultural, and racial inequalities [3],[7]. Racialized (i.e., visible minorities or people of color) and under-resourced immigrant families are most likely to face the heaviest burden from COVID-19 [8],[7]. They are most likely to experience financial insecurity and least likely to live in homes with access to backyards for their children or to be afforded the flexibility to work from home or the option to travel to work by car or via public transportation at less-crowded times. Racialized immigrants, in particular, are over-represented among the groups of essential workers in jobs that require direct contact with people or involve precarious conditions [7]. These challenges add stress to immigrant parents and their families. Additionally, regulations related to COVID-19 have meant that vital sources of informal and formal support that many immigrant families rely on, such as translation support and services, have been restricted or taken away entirely, thus increasing their risk of stress and burnout [8].

### 1.1. Study purpose

This paper focuses on the COVID-19 related changes and challenges experienced by immigrant parents living in apartment buildings in the Greater Toronto Area of Ontario Canada. It is based on a larger study that aimed to capture, from the points of view of immigrants living in apartment buildings, the physical-distancing challenges they faced and the successful strategies they used to maintain social connectedness during the COVID-19 pandemic.

## 2. Materials and methods

### 2.1. Theoretical approach

Our study was informed by an ecosystemic framework [9], which can help clarify how individuals are influenced by micro- (family), meso- (community), and macro- (society) level factors. Understanding the individual experiences and responses of immigrant parents to the pandemic must consider the multilayered systems that create inequity and inequality in basic need areas such as housing, employment, healthcare, and transportation that affect them [10]. For example, during the first six months (summer and fall of 2020) of the COVID-19 pandemic in Canada, meso- and macro-level factors and policymakers were primarily responsible for shaping decision-making surrounding COVID-19 restrictions such as school and workplace closures, which were then expected to be implemented by individuals and families. The ecosystemic framework helped reveal how some individuals and families have been disproportionately affected [10] by the COVID-19 restrictions related challenges of parenting during the pandemic.

### 2.2. Study design

This paper is based on a larger study that sought to capture how apartment-dwelling immigrants are affected by the pandemic, and the meso-level factors such as neighbourhood and city contexts and macro-levels factors such as provincial and federal guidelines related to the pandemic. The study used a qualitative interpretive descriptive method [11], which is widely applied to find practical solutions to real-life problems. This method also allows for flexible inquiry into the experiences of individuals [11]–[13]. Consistent with the principles of community-based research, the overall study design, including guidance for culturally, linguistically, and contextually appropriate approaches for pursuing participant recruitment, specification of the interview questions, seeking of informed consent, provision of honoraria, and conduct of interviews, was done in collaboration with our community partners, who have a deep understanding of the interests, concerns, and needs of immigrant communities.

After obtaining approval from the Ryerson Research Ethics Board (REB#2020-179), a purposeful sample of immigrants aged 18 years or older and living in apartment buildings in the Greater Toronto Area was recruited via word of mouth, email, social media, and through our connections in the city and with community partners who work with immigrants. Potential participants contacted (or were contacted by) the research assistants (RAs) to learn more about the study. Those interested in participating were provided a copy of the consent form prior to the interview. They were given the option to complete and return it to the RA prior to the interview or provide verbal consent at the time of the interview. Individual interviews were conducted between May and September 2020 on the phone or using a virtual platform (e.g., Zoom, Skype). Participants who did not have access to such technology were excluded.

Participants were interviewed using semi-structured interview questions that explored their experiences of the changes and challenges in their life related to the physical distancing measures, how they are coping with these changes and challenges, and the successful strategies used to maintain social connectedness. The interview questions were developed in consultations with our community partners. Interviews lasted about 30–45 minutes, on average. An honorarium ($30) was given to each person in consideration of their time in participating in the study.

Interviews were conducted in each participant's language of preference (e.g., Urdu, Spanish, Korean, and Arabic), by bilingual RAs. All interviews were audio-recorded with consent. Interviews conducted in a language other than English were translated into English and transcribed by the RA who conducted the interview. The interviews conducted in English were transcribed by an RA on the research team. Transcripts were read paragraph by paragraph, and coded by three members of the team to establish a coding system. After interviews were fully coded another iteration of analysis ensued with the help of several other team members to compare and contrast codes and to develop similar ideas into subthemes and themes, using thematic analysis [14].

Trustworthiness of the study was ensured through several strategies including interviewer triangulation (interviews were conducted by several interviewers); interviews conducted in different languages (to gain a broader and more comprehensive understanding of the topic by capturing opinions of participants of diverse ethnocultural and linguistic backgrounds); member-checking (with each participant during her/his interview, and with other study participants during subsequent interviews); and seeking community partners' feedback and reactions to the results and the interpretations of the results.

### 2.3. Study participants

In total, 72 immigrants participated in the larger study. Of these 50 participants were parents who are the focus of this paper. Of the 50 immigrant parents, the majority (n = 44; 88%) were mothers. Their ages ranged from 26 to 77 years, and most (n = 36; 78%) had two or more children. They described living in difficult conditions marked by confined and overcrowded apartments in high-rise buildings (n = 45) or subsidized housing (n = 5). About 40% of the participants had been in Canada for less than 10 years. Most reported experiencing financial insecurity due to unemployment—from before COVID-19 or as a result of the pandemic—or having only one partner trying to support the entire family by working in a low-income job. Table 1 provides additional demographic information.

**Table 1.** Socio-demographic characteristics of participants (n = 50).

	N	%
**Gender**
Men	6	12
Women	44	88
**Age**
26–35	9	18
36–45	12	24
46–55	12	24
>55	11	22.
no response	6	12
**Number of children**
1	13	26
2	22	44
3–5	15	30
**Number of people residing in household**
1–3	27	54
4–6	19	38
7+	4	8
**People living in the same house**
Children only	13	26
Spouse only	6	12
Children and spouse	28	56
Spouse, son, and daughter-in-law	2	4
Other	1	2
**Year of arrival to Canada**
Before 1985	2	4
1985–1994	6	12
1995–2004	15	30
2005–2014	11	22
After 2014	16	32
**Country of origin**
China	8	16
Pakistan	8	16
Bangladesh	6	12
Afghanistan	5	10
South Korea	4	8
Syria	4	8
India	3	6
Sri Lanka	3	6
Czech Republic	2	4
Philippines	2	4
Iraq	1	2
Nigeria	1	2
Russia	1	2
Slovakia	1	2
Somalia	1	2
**Current status in Canada**
Canadian citizen	35	70
Permanent resident	15	30
**Work status**
Did not work before COVID-19	27	54
Stopped working because of COVID-19	11	22
Currently working from home	6	12
Currently working as essential worker	4	8
Retired	1	2
Maternity leave	1	2

## 3. Results

Parenting has changed during COVID-19 in terms of parenting roles, responsibilities, and expectations. The following sections explore how immigrant parents experienced changes related to: dealing with changing relationships, coping with added burdens and pressures, living in persistent fear and anxiety, and rethinking lifestyles and habits.

**Figure 1.**
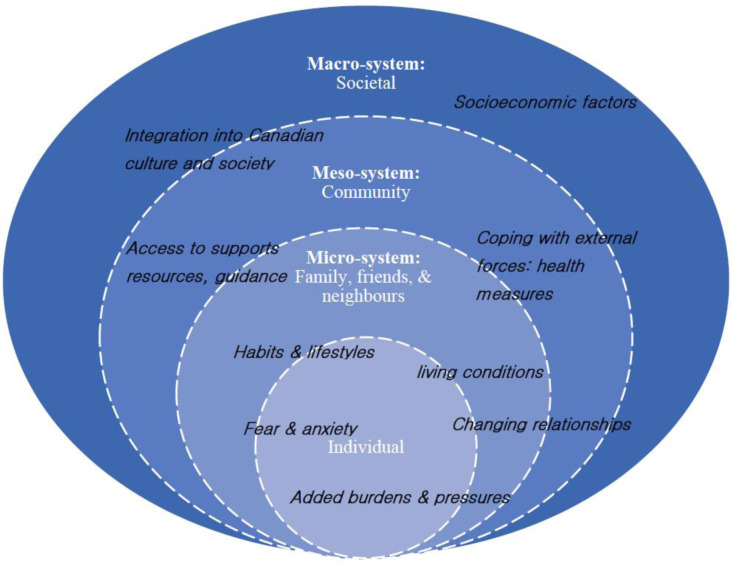
Parenting experience depicted using the ecosystemic framework.

### 3.1. Dealing with changing relationships

Participants reported that COVID-19 regulations of maintaining physical distancing changed family cohesion. Some reported a diminished sense of closeness within the family. For example, a father explained, “Physical distancing is a good thing to follow but it has separated us from our loved ones”. In contrast, some parents viewed having their children at home all the time as an opportunity to become closer and spend quality time with them:

“I spend time with them, and I relate to their experiences more now.”

“As a single mother, I have always been close to my daughter. During the pandemic, we do more activities like tea parties, so we feel even more connected. Before [the pandemic] it was go, go, go all the time.”

Some participants said that their children appreciated the extra time spent with their parents. For some parents, this provided a chance to connect their children to their cultural and linguistic roots:

“My children loved having me home all the time, and we watched Korean dramas on Netflix together so that I could teach my children about the Korean history and culture.”

Although they were worried about the pandemic, most parents demonstrated resilience and optimism. Some referred to growth in terms of parent-child relationships:

“During heated arguments while living and spending so much time together in a confined space, we learned to resolve arguments more diplomatically as we are stuck together instead of walking away or remaining bitter and resentful.”

“Being able to stay home with my family and give my child and husband time was a positive experience for me.”

However, some explained that spousal relationships, now lacking social and cultural supports combined with the inability to freely go outdoors, were negatively affected. Some participants noted that they had a designated spouse who went to work, and did grocery shopping and other “outside” activities such as going to the post office or taking garbage to the bins outside the apartment building, all of which potentially put them in closer proximity to others. Because of safety concerns for the rest of the family, this spouse was relegated to a different room in the apartment for periods as long as 4 to 6 months, a situation that created tensions between spouses, which, in turn, were noted as affecting their parenting styles.

### 3.2. Coping with added burdens and pressures

The closure of schools and childcare facilities placed sudden and enormous pressure on parents, especially mothers, to be fulltime parents, spouses, caregivers, and in some cases, full or part-time employees. The majority of mothers had no respite from any of these roles, responsibilities, and expectations:

“When kids were in school I had time for myself but not anymore and I have to deal with pressure from children all the time.”

“There are many changes in our lifestyle, all my family works from home, so I am busy a lot now, catering to them all the time and no time for myself.”

Some participants noted that the burden of being a mother and wife was magnified as they tried to maintain a “normal” home environment for children (and others) who are also constrained at home with each other often in small apartments for months. The participants noted that they had to be available to “entertain” or spend time with their children most of the time. Some participants remarked that they spent “every waking moment” thinking about and planning activities for their children that could take place within their small apartments to keep them busy. Participants who continued to work during the pandemic found this to be an impossible task.

A major burden identified by respondents that placed enormous pressure on them was related to the quality of their children's education during the pandemic. Due to physical distancing regulations implemented by the Canadian authorities, schools rapidly shifted to an online virtual format. Living in crowded apartments and sharing one computer was common and problematic for families with multiple school-aged children. In total, 75% of the participants had two or more children; 30% had three or more children. Some families did not have enough bandwidth to have multiple Zoom (Zoom Video Communications, a commonly used internet platform) classes and meetings at the same time. In addition, some parents did not speak English at all or sufficiently well enough to help their children learn. Others lacked computer or academic literacy to assist their children with virtual learning. Overall, most parents expressed difficulties in managing the various virtual learning expectations of the school boards, and the challenges were exacerbated for those with multiple children.

The perception that quality of education was compromised contributed to the cumulative stress of parenting. Many felt that online schooling was inadequate and that schools were inadequately-prepared. As a result, participants felt obligated to fulfill the double burden of serving as a teacher and parent—without appropriate support or preparation. One mother said:

“My children's schooling became a priority over everything else. This took up most of my time and energy.”

Not only did most participants believe that in-person schooling is more effective for student learning, but managing their children's disappointment of missing being in school and seeing their friends added to the challenging day-to-day tasks of parenting. Additionally, some participants had come to Canada in hopes of providing their children with better education. They saw the move to virtual learning as limiting their children's opportunities, for example, for developing English proficiency and becoming familiar with Canadian customs by interacting with peers. One mother reported that she had relied on a childcare facility to improve her son's English language skills: “My son does not speak much English. He is two years old, but all daycares are closed.” Overall, our study participants found the changes to their children's education to be extremely stressful and a hindrance to promoting their integration into Canadian mainstream culture.

### 3.3. Living with persistent fear and anxiety

Some participants said that living through the pandemic meant living with persistent fear, especially regarding their children's fate. One mother said, “I am scared for my children, for my children's future. We don't know what's going to happen. It's draining me mentally.” Overall, parents felt it necessary to continuously remind children about physical distancing, and expressed constant anxiety because their children could not remember or abide by the specific safety expectations and strategies. As one father said “Taking care of a toddler is more difficult because they do not understand the situation.” Mothers, in particular, felt that they had to be constantly vigilant, which they found mentally and physically exhausting. Some were frustrated that their children did not always adhere to public health measures to protect themselves from COVID-19, but they were also aware of the absurd expectations being placed on young children. Some commented about these “no-win” circumstances. For example, one mother told us:

“Sometimes I don't even know what to do. I can't scold them or yell at them. Look at the situation. You see so many people getting infected everyday. When you go out, you're scared of getting the germs. When you don't go out, you're scared they're going to go crazy from being trapped or become unhealthy.”

Children's health, and particularly their mental health, was a relentless concern for parents who were worried that their children's mental health would “deteriorate” and that lockdown could have “negative impacts in their future.” As one mother told us:

“My child can't go outside and often feels depressed staying inside as she loves being around people. I worry that this maybe affecting her developmentally, as my child is very young and this age needs socialization and exploring the outside environment.”

While acknowledging the importance of technology in keeping their children occupied, connected, and learning, all participants referred to being “very worried” about children's “constant” use of technology during the lockdown, and the potential negative consequences that this could have on their physical health as well. Having to use Zoom or Skype or other platforms for virtual schooling added to this worry. The following quote from a mother captured the reality expressed by all parents:

“Kids are home and using electronic devices all the time, it's very hard to make them do physical activities and keep them occupied.”

Many questioned the impact that 8 to 10 or more hours of long-term exposure to electronic devices on their children's eyesight, hearing, and brain. These fears are heightened for some because of the immigration context of their lives in Canada. One mother explained:

“I often feel scared that my child will become sick and if so as a first-generation immigrant to Canada I don't think I have enough support to take care of her.”

Overall, parents were anxious and afraid about the uncertainty surrounding the pandemic and related restrictions, and their short- and long-term physical, mental, and emotional health consequences on their children.

### 3.4. Rethinking lifestyles and habits

Parents noted that their lifestyles and daily habits had changed due to the pandemic. Some of these changes are captured in the excerpts below:

“Sleeping habits are a big challenge. My daughter used to go to sleep around 7 or 8 pm. And then she wakes up at 6 or 7. But that has changed for her (...) so I sleep at 1 or 2 am. She still wakes up early. So her sleep is affected and my sleep is affected.”

A single mother noted that she had to leave her kids at home alone at times because when she did take them with her on errands (e.g., grocery shopping) she received negative “looks or comments from other people in the store.” Due to COVID-19 restrictions, she could not seek help from her other family members, neighbours, or friends who provided such help prior to the pandemic.

Some participants commented on the impact of the COVID-19 restrictions on their daily activities and habits that kept their food and other expenses at a manageable level. As one participant explained:

“I cannot shop around for bargains anymore because I don't want to stay in long lineups or take the bus or the subway because I am worried about spending too much time around other people and...and getting the virus. Some people are not wearing masks and don't do social distancing.”

These changes have financial consequences on their lives. Other changes to their habits included the extra precautions that they took when interacting with their children:

“When my husband goes outside even for a little while and comes home, I make him take a shower before bringing our child near him.”

“Although me and my daughter would still eat together, we each use different plates and other tableware.”

More drastic changes to their lives included decisions parents made about their own work and education. One participant noted being afraid of going back to work: “I worry about infecting my daughter if I were to go back to work.” While another noted that her husband had “dropped out of college to take care of our kids.”

Despite the added stressors that many of these changes added, not all changes were perceived negatively. Some parents referred to positive changes such as improved family dynamics:

“Our life has changed totally, before the pandemic life was very busy everything seemed [to be] going very fast and there was a lot to do, but now the whole family has to stay home, and things have slowed down considerably.”

“I made healthy meals for my family and home schooled two of my three children. I created a daily routine for them to follow, which included regular walks in the park nearby.”

Some of these participants even wondered whether these changes could be maintained if and when life returns to “normal”.

## 4. Discussion and implications

Our findings provide insights into the parenting experiences of immigrants during COVID-19. At the beginning of the pandemic (March 2020), public health guidelines rapidly and frequently changed as new knowledge emerged, leading to changes in federal, provincial, and municipal level health, education, and economic policies that understandably exacerbated public uncertainty and anxiety. Most of these policies ignored the realities of families living in apartment buildings where physical distancing is a challenge. For many Toronto immigrant families living in such spaces, the physical distancing required by policies—and especially separate spaces for children and older persons—was extremely difficult as they tried to navigate crowded apartment buildings, shared elevators, and laundry rooms. Moreover, many take public transport, live in multi-generational and/or multi-family households, and work in essential services.

Most participants did not speak English fluently and were not very comfortable with technology and the virtual world beyond basic use. Many were not very familiar with the Canadian education system and feared the disruptions caused by the pandemic would affect their children's education and futures. Many immigrant parents came to Canada to provide their children with a better education, which is often seen as a source of upward mobility. During COVID-19, children have been expected to learn from home in an online format, but the rapid shift to virtual delivery of education in Ontario has been underpinned by normative assumptions of families' social locations and privileges, assuming familial access to technology and internet services. The experiences shared by parents in this study reflect other research findings about the digital divide and equity in access to resources [15]–[17]. Our participants noted that the burden to facilitate virtual delivery of education fell primarily on parents, not all of whom have the skills, knowledge, or time to provide this support. They found online learning unsatisfactory: many viewed it as hindering their children's learning and worried that it would affect their socialization, English language acquisition, psycho-social development, and overall acculturation to Canada.

Beyond the impact on education, COVID-19 restrictions and government-mandated public health measures such as closure of schools, childcare, and other non-essential services curtailed access to formal and informal support networks. Due to limited English language proficiency and social networking, newcomer immigrant parents already lack social support apart from informal support from their ethno-cultural communities. The loss of informal social connections can amplify factors contributing to parental stress through increased social isolation, inability to access supportive and educational services, and economic difficulties [18]. Given the intimate nature of parent-child relationships, parental experience, particularly parental stress, may influence a child's experiences with the pandemic and overall wellbeing [1].

For mothers who were engaged in paid employment outside the home prior to the pandemic, work provided a sense of freedom and time away from home and family responsibilities. Even those who were not employed in paid work prior to the pandemic noted that having to cater to their children, husbands, and other family members who were always at home meant that they were not able to find any time for themselves. Just over 25% of the study sample did not live with their spouse, and managing these responsibilities were near impossible for them while facing financial constraints and with almost no outside help. The disproportionate impact of the pandemic on families with lower socioeconomic status reflects a lack of understanding about how macro-level factors such as policies play out for immigrants at the meso (community) and micro (family) levels. For example, parents living in small apartments had trouble finding and setting up activities for their children given their confined space; they also struggled with sharing limited technology devices and limited or unreliable internet services, highlighting the need to address digital inequity in supporting students from low socioeconomic backgrounds.

Participants were worried about the impact of school closures on their children's health and wellbeing. In addition to the loss of social and emotional space for development and social and cultural integration, they noted that children were less physically active, had longer than usual screen time, and had irregular sleep patterns; some noted that children had less healthy diets, resulting in weight gain and a loss of cardiorespiratory fitness. The psychological impact of stressors such as prolonged duration at home, fears of infection, frustration and boredom, inadequate information, lack of in-person contact with classmates, friends, and teachers, lack of personal space at home, and family and financial loss, all appeared to contribute to tensions in relationships between parents and children.

Overall, participants referred to changes in sleeping patterns, anxiety, fear, frustration, and worry. Mental health resources that are sensitive and responsive to the unique needs and contexts of immigrant families are needed. Supportive strategies could include community-oriented approaches, for example, online community group discussions and parenting groups [7]. This kind of strategy should include interpreters and translators, and may require creativity in order to respond to the varying abilities to access the internet, communication devices, and levels of digital literacy. It will be important to build on the resilience of immigrant families and communities to help them adjust and adapt as the pandemic progresses; it may be beneficial to create a safe space outside the family, especially for mothers. For example, community networking is known to facilitate positive parenting and positive outcomes for children [8]. Immigrant parents obtain most support from their ethnic communities, but broadened networks would allow parents from various backgrounds to share their experiences of living through the pandemic. Additionally, provincial, federal, and local governments should provide targeted information in addition to instrumental, financial, and health-related resources and support to immigrant communities.

The pandemic and the unintended consequences of macro- and meso-level policies have compounded the already considerable health inequities immigrant families experience due to poor housing, unsafe neighbourhoods, lack of access to services, limited income due to underpaid and precarious work, discrimination, and racism [10],[19]–[21]. The lack of availability of resources to support immigrant parents requires immediate attention [1] because it can affect the mental and physical health of parents, parenting, and parent-child relationships.

## 5. Conclusions

COVID-19 has not only increased pre-existing parental responsibilities but has created many new demands on parents. Contributing to the negative experiences reported by parents in our study is the exacerbation of pre-existing inequalities among immigrants that have been heightened by the pandemic. The situation of many families in this study has become worse due to reduced informal social support, lack of familiarity with the educational system, and lack of ready access to internet, computer, as well as indoor/outdoor space to keep their children socially-connected or occupied while physically distancing. These numerous challenges related to the pandemic have created new stresses, anxieties, fears, and frustrations for them that can affect their own well-being and that of their children. Further, the gendered impact of the pandemic has been enormous on mothers who have been forced to take on most of the family burden related to COVID-19. Yet, they have shown significant resilience in ensuring their children's physical, emotional, and mental health and wellbeing. Building on their resilience, immediate action must be taken to help vulnerable parents, mothers in particular, in order to prevent the long-lasting negative effects on them and their children.
